# School participation of autistic youths: The influence of youth, family and school factors

**DOI:** 10.1177/13623613231225490

**Published:** 2024-02-04

**Authors:** Boya Li, David Heyne, Anke Scheeren, Els Blijd-Hoogewys, Carolien Rieffe

**Affiliations:** 1 Leiden University, The Netherlands; 2 Delft University of Technology, The Netherlands; 3 Deakin University, Australia; 4 VU University Amsterdam, The Netherlands; 5 INTER-PSY, The Netherlands; 6 University of Groningen, The Netherlands; 7 University of Twente, The Netherlands; 8 University College London, UK

**Keywords:** autistic traits, autistic youth, physical environment, school participation, social environment

## Abstract

Many autistic youths experience restricted school participation. The present study investigated the influences of youth, family and school factors on autistic youths’ school participation. Parents of 200 Dutch autistic youths (age range: 4–16 years, M_age_ = 12.23 years, SD_age_ = 2.93 years) filled in questionnaires in a national survey for autistic individuals, reporting the school participation, age and autistic traits of their child, parents’ education level and self-efficacy for supporting their child’s schoolwork, and the impact of problems their child experienced with the physical and social environments of the school. Multivariate linear regression analysis using imputed data revealed that among the six predictor variables, only the impact of problems autistic youths experienced with the physical environment of school was negatively associated with their school participation. This study provided support for the essential role of the school environment in predicting autistic youths’ school participation, indicating that problematic aspects in the school environment could have a greater impact on autistic youths’ school participation than youth factors or family factors. This highlights the need to create a more accommodating environment at school, where autistic youths can participate easily and comfortably.

**Lay abstract:**

School-aged youths have a basic human right to participate in educational and recreational activities at school. Yet, autistic youths are at high risk of being excluded from school and from school-based activities. It is important to understand how this occurs, to ensure that all autistic youths have opportunities to participate in school activities that are equal to the opportunities of their non-autistic peers. The present study investigated multiple influences on the school participation of autistic youths, including youth factors (age and autistic traits), family factors (parent education level and parental self-efficacy for supporting their child’s schoolwork) and school factors (the impact of problems autistic youths experienced with the physical and social environments of school). Using an online survey, we gathered the views and experiences of the parents of 200 autistic youths aged between 4 and 16 years, in the Netherlands. We found that among the factors, only the impact of problems that autistic youths experienced with the physical environment of school was associated with their school participation. In particular, autistic youths who experienced greater difficulties with the physical environment of school had lower levels of school participation. Our findings highlight the pressing need to modify school environments to better accommodate the needs of autistic youths so that they can participate easily and comfortably.

For school-aged youths, attending school provides them with the opportunity to participate in educational and social activities. School participation lays the foundation for knowledge acquisition, equipping youths with the skills required to attain financial self-sufficiency and lead independent lives in adulthood (Balfanz, 2016). Furthermore, for many youths, school participation fosters a sense of belonging, facilitates opportunities for social learning, and contributes to a better quality of life (Allison & Attisha, 2019; Ramstetter et al., 2010).

Nevertheless, youths diagnosed with autism are often at risk of exclusion from school and school-based activities (Law et al., 2007; Mattson et al., 2022). When these youths do attend school, participation can lead to adverse effects, such as sensory overload, physical exhaustion, and mental distress. Autistic youths are often unfairly held accountable for the exclusion they experience (Edey et al., 2016; Keating & Cook, 2020). According to the biopsychosocial model proposed by the International Classification of Functioning, Disability and Health (ICF; World Health Organization, 2001, 2007), an individual’s activities and participation are the outcomes of interactions between the individual’s health condition, personal factors, and environmental factors. Therefore, when examining the school participation of autistic youths, it is important to consider the influence of intrinsic personal factors (i.e. youth characteristics) as well as extrinsic environmental factors such as those associated with a youth’s family and school.

## Autistic youths’ school participation

School-aged youths have a basic human right to participate in school-related educational and recreational activities, whether these occur inside the classroom, on the school grounds, or outside the school setting such as school excursions. However, there is wide documentation of restricted school participation among autistic youths (Brede et al., 2017; Falkmer et al., 2015; Hatton, 2018).

First, compared to the general population of school-aged youths, autistic youths have higher rates of absenteeism and are at higher risk of dropping out of school (Adams, 2022; Munkhaugen et al., 2017). For example, in the Netherlands, the unenrolment rates of children of primary-school age and of secondary-school age in 2016 were 2.3% and 0.8%, respectively (CEIC Data, 2021), whereas a national survey conducted in 2022 reported that the unenrolment rate among autistic youths under 16 years of age was 11.7% (Netherlands Autism Register, 2022). A UK survey of 486 autistic youths enrolled in a school revealed that 43% missed 10% or more of school time (i.e. persistent absence) during a 1-month period (Totsika et al., 2020). This was considerably higher than the 11% of youths with persistent absence as reported for the general youth population in the UK (Department for Education, UK, 2019).

Second, when autistic youths do attend school, their mere physical presence there does not guarantee them full access to school activities. Keen et al. (2023) reported that autistic youths find it more challenging than non-autistic youths to follow the school curriculum, they feel less motivated to participate in learning activities, they experience more difficulty and stress in completing school tasks, and they are more prone to academic underachievement. Autistic youths also face great challenges in the social domain at school. Much research attests to the fact that autistic youths, compared to non-autistic peers, are more subject to social exclusion and bullying (Kaljača et al., 2019; Locke et al., 2016) and more often report loneliness in school (Chang et al., 2019; Deckers et al., 2017).

The lower rate of school participation among autistic youths is a cause for concern because school participation has been shown to be crucial for fostering development, but this is the case only when schools create an autism-friendly environment. A comprehensive literature review conducted by the European Agency for Special Needs and Inclusive Education (2018) reveals that youths with disabilities who receive their education in inclusive settings tend to perform better both academically and socially. They also have a higher likelihood of securing employment and report greater overall life satisfaction than those who did not participate in school education or who were educated in segregated settings. For autistic youths, exclusion from school or from activities within school can have acute and long-term adverse effects on their mental, social, and economic wellbeing (Brede et al., 2017; Martin-Denham, 2022; Zablotsky et al., 2013). Therefore, it is imperative to understand the factors that facilitate autistic youths’ school participation and identify the obstacles that hinder their attendance and participation in school activities.

## Youth factors

Age is a salient personal factor frequently considered in the examination of youths’ participation in activities (Chien et al., 2017). Concerning school attendance, older youths in the general population are more susceptible to experiencing emotional distress about going to school and exhibit higher rates of absenteeism than younger children (Heyne, 2023). This age-related susceptibility may be associated with the new challenges presented by the transition from primary school to secondary school, such as encountering a greater variety of subjects, an increased number of exams (Anderson et al., 2000; Curson et al., 2019), moving between lessons, and navigating a larger and more complex schoolyard layout (Bagnall et al., 2021; Mumford & Birchwood, 2021). Furthermore, as youths grow older, social interactions at school become more intricate, requiring advanced social skills like perspective-taking and negotiating personal boundaries without jeopardizing relationships with others (Collins & van Dulmen, 2006; Semrud-Clikeman, 2007).

The increasingly demanding educational tasks and increasingly complex social environment at school may be more challenging for autistic youths than for non-autistic youths. For example, Makin, Hill and Pellicano (2017) found that when transitioning from primary education to secondary education, autistic youths experienced more severe anxiety, and they struggled more with adjusting to a new school environment than non-autistic youths (see also Mandy, Murin, Baykaner, Staunton, Hellriegel, et al., 2016). In addition, the loneliness and social exclusion experienced by autistic youths appear to increase with age (Ratcliff et al., 2018; Simpson et al., 2019). While autistic youths’ social skills also improve with age (Gray et al., 2012; Seltzer et al., 2004), their style of socialization and communication may never fully align with the way most people socialize in the predominantly non-autistic world. Differences between autistic and non-autistic styles of communication may even expand over time (Picci & Scherf, 2015), which could increase the risk of autistic youths being socially excluded because many non-autistic youths become less accepting of others’ differences, and they become more selective in their friendships as they grow older (Aboud et al., 2003).

Another personal factor that could potentially influence autistic youths’ school participation is the level of autistic traits. The aforementioned complexities of school participation may be more challenging for autistic youths with more accentuated autistic traits, relative to those with ‘milder’ autistic traits. Indeed, youths with higher levels of autistic traits were found to have greater difficulty adjusting to the often-unpredictable school environment, making plans for school tasks, maintaining attention to teacher instructions, and overcoming sensory hyperarousal or hypoarousal, compared to autistic youths with mild symptoms (Chiang et al., 2018). Higher levels of autistic traits are also associated with greater challenges in processing social information and in establishing and maintaining social relationships (Hilton et al., 2010; Hsiao et al., 2013).

## Family factors

The extent to which families support autistic youths’ engagement and success at school will likely influence these youths’ school participation. Two family-related factors of interest are socioeconomic status and parental self-efficacy.

First, families’ socioeconomic status might be positively associated with autistic youths’ school participation. Research indicates that autistic youths from affluent families are more likely than youths from families with a lower socioeconomic status to receive a timely diagnosis at a young age, have greater access to social services and professional help, display fewer behavioural and mental health problems, and have better school performance (Carr et al., 2016; Kelly et al., 2019; Robinson & Weiss, 2020).

The second factor of interest is parental self-efficacy, which refers to parents’ confidence and perceived competence in parenting. Parents’ perceived efficacy in assisting their child with schoolwork has been found to benefit school performance among youths in the general population (Hoover-Dempsey et al., 2005; Jones & Prinz, 2005). Parents with higher self-efficacy have stronger motivation to engage in their child’s education, and they are more persistent in overcoming challenges and obstacles to supporting their child’s school success, relative to parents with lower self-efficacy (Benner et al., 2016; Hoover-Dempsey et al., 2005). The aforementioned associations between parental self-efficacy and youths’ school performances have been established based on research conducted within the general population of school-aged youths, not specially within the context of youths with autism. Raising an autistic child in a predominantly non-autistic world presents parents with unique challenges and substantial stress. Research among the parents of autistic children points to a negative association between parental stress and perceived self-efficacy in parenting (Chen et al., 2021; Kuhn & Carter, 2006; May et al., 2021). Moreover, parental self-efficacy is lower among the parents of autistic children relative to parents without an autistic child (Karst & Van Hecke, 2012). To date, little is known about the extent to which parents’ perceived self-efficacy contributes to autistic youths’ school participation. The study by Bar, Shelef and Bart (2016) is the only one we are aware of that examined maternal self-efficacy in relation to autistic pre-schoolers’ participation in daily activities, yielding moderate to strong positive associations between maternal self-efficacy and the number of activities autistic pre-schoolers participated in, and the pre-schoolers’ level of enjoyment.

## School factors

The challenges autistic youths experience with respect to school participation often arise from a mismatch between their autism-associated characteristics and the school environment which is commonly designed to meet the needs of non-autistic youths (Bailey & Baker, 2020).

It is well established that autistic individuals process cognitive and sensory information differently from non-autistic individuals (Ben-Sasson et al., 2009; Tomchek et al., 2014). However, the characteristics and needs of autistic youths are rarely considered in the architectural design of schools (Mostafa, 2008; Rieffe & Koutamanis, 2023). For many autistic youths, it takes a lot of energy to understand and assimilate the physical environment surrounding them, which is designed to serve the needs of non-autistic youths (Vázquez & Torres, 2013). For example, one characteristic of autism is having difficulty dealing with changes in daily routines (American Psychiatric Association, 2022). Nevertheless, it is common for youths in secondary school to be expected to move from one space to another for different educational activities, often with all students moving simultaneously. It can be extremely stressful for some autistic youths to find their way through overcrowded hallways with poor acoustics. Such challenges can lead to exhaustion and may deter them from participating in school activities or socializing with their peers (Rieffe & Koutamanis, 2023). Many autistic youths have hyper- or hypo-sensitivities. However, the physical environments of schools often contain sensory stimuli, which trigger sensory overarousal or create perception difficulties for autistic youths (Martin, 2016). An unaccommodating sensory environment at school may also provoke avoidance behaviours and lead to non-attendance among autistic youths (Ghazali et al., 2019; Mostafa, 2014).

An unsupportive social environment at school may also contribute to reduced school participation among autistic youths (Kapp, 2018; Ochs et al., 2001). Autistic youths experience a low sense of belonging at school and high rates of social rejection and bullying when the school culture is characterised by a low acceptance of diversity and individual differences, when there is a lack of understanding of autism, when teachers lack competence in instructing autistic students, and when there is inadequate adaptation of teaching and curriculum for autistic students (Anderson, 2020; Danker et al., 2019a, 2019b; Hernández González et al., 2022; Symes & Humphrey, 2011). In contrast, participation becomes less stressful and more attractive for autistic students when the school culture focuses on equality and embraces individual differences (Goodall, 2015), when autistic youths are expected and invited to participate in school activities, and when they are provided with opportunities and supports to participate (e.g. incorporating the special interests and strengths of autistic youths into the design of school activities) (Hodges et al., 2020; L. K. Koegel et al., 2012; R. L. Koegel et al., 2012; Rotheram-Fuller et al., 2010).

## The present study

Attending school and participating in educational and social activities within the school environment represent a crucial aspect of daily life for autistic youths. However, the benefits of school participation for autistic youths are contingent upon the inclusivity of the school environment and the meaningfulness of school activities (European Agency for Special Needs and Inclusive Education, 2018; Falkmer et al., 2012). Placing autistic youths in a school environment that does not consider their needs and capacities can lead to stress, fatigue, and limited school participation (Bailey & Baker, 2020; Brede et al., 2017; Falkmer et al., 2015). Initial investigations of autistic youths’ school participation focused largely on youth-related characteristics such as cognitive and social abilities. Recently, there has been increasing attention to the impact of the environment on autistic youths’ school participation. Nonetheless, there is little quantitative research on the associations between environmental factors and autistic youths’ school participation, and no study has simultaneously explored youth and environmental influences on school participation. To address this gap, the current study employed the ICF model to explore the role of youth factors (age, autistic traits), family factors (parents’ education level and parental self-efficacy) and school factors (the impact of problems with the physical and social environments of schools) in predicting school participation among autistic youths aged 4 to 16 years, in the Netherlands. Based on previous findings related to autistic youths, we expected autistic youths’ age and the level of autistic traits to be negatively associated with their school participation. We expected parents’ education level and parental self-efficacy to be positively associated with autistic youths’ school participation. Furthermore, we expected the impact of problems experienced by autistic students with the physical and social environments of school to be negatively associated with autistic youths’ school participation.

## Methods

### Participants and procedure

This study is part of a research project (i.e. the Inclusive School Environment (ISE) Project) conducted at Leiden University in the Netherlands, which investigates ways to promote inclusiveness in the school environment for youths with an autism diagnosis. Data for the current study were collected in the Spring of 2020 through collaboration with the Netherlands Autism Register (NAR), a research organization managing a longitudinal cohort of Dutch individuals with autism. Data pertaining to the current study were derived from parent responses. Parents provided written informed consent to participate in the study. The ISE project was approved by the Psychology Research Ethics Committee of Leiden University (V2-2428-data 04 June 2020; V3-2685-date 24 October 2020). There was no community involvement in this study.

The sample of this study included 200 autistic youths aged between 4 and 16 years (M_age_ = 12.23 years, SD_age_ = 2.93 years). In the Netherlands, children go to primary school from 4 years of age and secondary school from around 12 years of age. According to parents, of these 200 youths, 18 (9%) were not enrolled at school at the time of data collection (i.e. spring, 2020), and there was one youth (1%) whose parent did not provide information about his or her school attendance. Among the remaining 181 youths, 64 (32%) were in mainstream education, 109 (55%) were in special education, and the parents of 8 (4%) youths did not provide information regarding the type of education their child was following. As commonly observed in autism research, our sample showed a biased sex distribution, with a higher percentage of male autistic youths than female autistic youths: N_male_ = 158 (79%), N_female_ = 42 (21%). In addition, our sample primarily comprised youths with IQ scores above 70 (N = 151, 84%), while only 16% of the youths (N = 29) had IQ scores of 70 or lower.

### Measures

Information about youth characteristics (i.e. age and autistic traits), youths’ school attendance (i.e. whether attending school and the type of school), and the education level of parents was collected through an online survey distributed by the NAR. For this study, three new questionnaires (see the content of the three questionnaires in Supplemental Table 1) were added to the survey to collect information on autistic youths’ school participation, parental self-efficacy, and the impact of problems with the school environment. Parents of youths who were attending schools were instructed to keep the current school of their child in mind when answering the questions, and parents whose child was currently not enrolled at school were instructed to keep the previous school of their child in mind.

The three added questionnaires were originally designed in English. Dutch adaptations were prepared as follows. First, two bilingual researchers fluent in Dutch and English translated the questionnaires from English to Dutch, working independently on their forward translations. Next, the two researchers compared their translations and resolved differences through discussion. A third bilingual researcher then examined the translated items and approved the translation. The research team of the ISE project evaluated the questionnaires and made some further adjustments, including editing the wording of items, adding information to items, and adding new items. For example, ‘It is difficult to see or to hear important information at school’ was added to the items measuring problems with the physical environment of school, and ‘Crime or violence at school’ was added to the items measuring problems with the social environment of school.

#### School participation

The scale measuring autistic youths’ school participation was translated and adjusted from the Child and Adolescent Scale of Participation (CASP; Bedell, 2011a). The CASP consists of four sections, asking parents to evaluate their child’s home participation, community participation, school participation, and home and community living activities. The current study used the scale measuring school participation. Specifically, parents reported the extent to which their child participated in activities at school relative to their classmates. Parents rated each item on a 3-point scale, from ‘1 = full participation’ to ‘3 = no participation’. For data analyses, responses were reverse coded so that higher scores indicated more school participation. Following the study by Bedell (2011a), a total score was calculated by summing the score for each item, dividing by the maximum possible score of all items together (i.e. 24) and multiplying by 100. The internal consistency of this scale for our sample was good, α = 0.81.

#### Youth age and autistic traits

Based on the parent’s report of their child’s date of birth, we calculated the age of the youths in the Spring of 2020. Youths’ level of autistic traits was measured via the short version of the Autism-Spectrum Quotient (AQ-Short; Hoekstra et al., 2011). The AQ-Short consists of 28 items covering five areas, including social skills, routine, attention switching, imagination, and number and patterns. Parents rated the items on a 4-point Likert-type scale, from ‘1 = definitely agree’ to ‘4 = definitely disagree’. A higher total score indicates a higher level of autistic traits. The AQ-Short showed good internal consistency for our sample, α = 0.83.

#### Parents’ education level and self-efficacy

Parents reported their highest obtained education level on a 3-point scale: 1 = no/elementary education; 2 = middle/high school education; 3 = college/university education. For data analysis, the mean score of the father’s and mother’s highest education level was calculated. Parents also filled in a questionnaire adapted from the questionnaire ‘Self-Efficacy for Helping the Child Succeed in School’ (Hoover-Dempsey et al., 1992; Walker et al., 2005). The adapted version comprised 11 items with a 5-point Likert-type scale, from ‘1 = definitely disagree’ to ‘5 = definitely agree’. Items that are negatively formulated were reverse scored to derive the total score, with a higher total score indicating higher self-efficacy. The internal consistency for the current sample was good, α = 0.80.

#### School environment

The Child and Adolescent Scale of Environment (CASE; Bedell, 2011b) consists of 18 items, asking parents about the impact of problems their child experienced with the environment at home, at school, and in community. Based on the CASE, we derived a four-item scale to measure the impact of problems youths experienced with the physical environment of school (e.g. ‘It is difficult to reach things and places’) and a five-item scale to measure the impact of problems experienced with the social environment of school (e.g. ‘There is a lack of support or encouragement at school (e.g. due to teachers or classmates).’). Each item was rated on a 3-point scale, namely ‘1 = no problem, 2 = little problem, and 3 = big problem’. Following the study by Bedell (2011b), we calculated the total score for each scale and divided it by the maximum possible score for that scale, then multiplied by 100. Higher scores indicate a greater impact of problems experienced with the school environment. The internal consistency for the scale ‘Impact of problems with the physical environment of school’ was acceptable, α = 0.61. The internal consistency for the scale ‘Impact of problems with the social environment of school’ was good, α = 0.80.

### Statistical analyses

IBM SPSS Statistics version 27 was used for data analyses. First, we conducted correlational analyses to explore the relations between the variables. Before the analyses, we checked the assumption of normality. Not all variables had normally distributed data. Therefore, the Spearman’s rank-order correlation test was used. Next, we conducted a multivariate linear regression analysis to test the hypotheses about the associations between the predicting variables (i.e. youth, family, and school factors) and autistic youths’ school participation. All predicting variables were entered in the regression model, using the ‘Enter’ method. Before the analysis, we checked the assumptions for the regression analysis, including homoscedasticity, multicollinearity, and normal distribution of residuals. The data inspection raised no concerns regarding these assumptions.

#### Multiple imputation for missing values

When all variables were entered into the Little’s MCAR (missing completely at random) test, the result was significant (χ^2^ = 280.82, *df* = 158, *p* < 0.001), indicating that the data were not missing completely at random. A close inspection of the missing values revealed that the variable ‘autistic traits’ had the highest missing rate. Out of the 200 youths, the parents of 67 (33.5%) youths did not fill in the AQ-Short. This was because the national database did not administer the AQ-Short to parents who stated that their child had an intellectual disability, as the questionnaire was deemed less suitable for this subgroup. When we removed ‘autistic traits’ from Little’s MCAR test, the result showed that the missing values among the remaining variables were missing completely at random (χ^2^ = 70.37, *df*= 63, *p* = 0.245). Moreover, parents of 42 autistic youths (21%) did not provide information regarding their children’s school participation, and 55 parents (27.5%) did not report on their perceived self-efficacy. For the counts and percentages of missing values for the study variables, please refer to Supplementary Table 2. We took the following two steps to deal with missing values.

First, we compared the characteristics of youths with complete and missing data (Sterne et al., 2009). Compared to youths with complete AQ-Short data, youths without AQ-Short data were younger (*t*(74.82) = −2.12, *p* = 0.037), had a higher proportion of boys (χ^2^ = 4.96, *p* = 0.026), had a higher percentage of participants with an IQ score below 70 (χ^2^ = 48.18, *p* < 0.001), and had parents who reported having lower self-efficacy (*t*(137) = −3.11, *p* = 0.002). Possibly, there was a selective attrition of participants with a high level of autistic traits because autistic traits tend to be more pronounced among autistic boys than among autistic girls (Mandy et al., 2012; Van Wijngaarden-Cremers et al., 2014) and among those with an intellectual disability than among those without (Hoekstra et al., 2009; Nishiyama et al., 2009).

Compared to youths with complete school participation data, those with incomplete school participation data were younger (*t*(53.81) = 2.45, *p* = 0.018), and their parents had a lower education level (*t*(187) = 2.3, *p* = 0.023). Furthermore, among youths with incomplete school participation data, a higher percentage were not enrolled in school at the time of data collection, and a lower percentage were enrolled in mainstream education (χ^2^ = 11.78, *p* = 0.003).

Compared to youths whose parents reported on self-efficacy, youths whose parents did not report this information had lower levels of school participation (*t*(53.81) = 2.45, *p* = 0.018) and more often had an IQ below 70 (χ^2^ = 11.76, *p* < 0.001). In addition, a higher proportion of these youths did not attend school, and a lower proportion of them attended mainstream education (χ^2^ = 15.67, *p* < 0.001).

The comparisons between youths with and without missing data suggest that youths with missing data were likely to be in more challenging situations, such as having more pronounced autistic traits, lower IQ, and having parents with lower self-efficacy.

We applied the multiple imputation technique (MI) to replace the missing values by imputed values, which were sampled from their predictive distribution based on the existing values from the dataset (Azur et al., 2011). We created 10 imputation sets of the study variables and other variables in the dataset, such as participant sex, information about diagnosis, household income, AQ-Short scores for subconstructs (e.g. social skills, imagination), and the psychosocial functioning of autistic participants (Graham, 2009). Next, we re-ran the correlation and regression analyses using the imputed dataset. In the following section, we report both the pooled results and the results using the original data (i.e. data before applying the MI).

## Results

### Results from the original data

#### Correlations between study variables

School participation was positively associated with age (*r* = 0.16, *p* = 0.045) and negatively associated with the level of autistic traits (*r* = −0.23, *p* = 0.017). Furthermore, school participation was negatively associated with the impact of problems youths experienced with the physical (*r* = −0.43, *p* < 0.001) and social environment of school (*r* = −0.33, *p* < 0.001). In addition, there was a negative association between parents’ education level and youths’ age (*r* = −0.15, *p* = 0.038), a positive association between the impact of problems experienced with the social environment of school and autistic traits (*r* = 0.18, *p* = 0.039), and a positive association between the impact of problems experienced with the physical and the social environments of school (*r* = 0.69, *p* < 0.001).

To account for multiple comparisons, we employed the Holm-Bonferroni method to adjust the *p* values (Holm, 1979; Hommel, 1988). First, the *p* values were arranged in ascending order. Next, for each *p* value, we tested whether *p*_k_ < α/ (*m* + 1 – *k*), where ‘*m*’ is the total number of *p* values, and ‘*k*’ is the sequence number of the *p* value that is being tested. Following the Holm-Bonferroni correction, the associations between school participation and the level of autistic traits, between parents’ education level and youths’ age, and between the impact of problems experienced in the social environment and the level of autistic traits no longer retained statistical significance. See Table 1 for the descriptive statistics and correlations of the study variables.

**Table 1.** Descriptive statistics and correlations (using Spearman’s rank-order correlation analyses) among study variables.

	Mean	SD	1	2	3	4	5	6
1. School participation	82.94	22.48	–	–	–	–	–	–
2. Youths’ age	12.23	2.94	0.16*	–	–	–	–	–
3. Youths’ autistic traits	81.78	10.46	−0.23	−0.08	–	–	–	–
4. Parent education level	2.37	0.59	−0.09	−0.15	−0.17	–	–	–
5. Parental self-efficacy	38.14	6.70	0.13	−0.02	0.02	0.04	–	–
6. Impact of problems with the physical environment	45.73	14.33	−0.43**	0.07	0.14	−0.06	−0.10	–
7. Impact of problems with the social environment	60.79	22.28	−0.33**	0.06	0.18	−0.008	−0.15	0.69**

SD: standard deviation.

* *p* < 0.05; **p < 0.001.

#### The regression model predicting school participation

Table 2 shows the outcomes of the multivariate regression analysis. The model with all the predicting variables explained 45% of variance in autistic youths’ school participation. Autistic youths’ age was positively associated with autistic youths’ school participation (*t* = 2.89, *p* = 0.005), whereas the greater impact of problems experienced with the physical environment of school contributed to predicting less school participation among autistic youths (*t* = −5.76, *p* < 0.001). The other factors (i.e. youths’ autistic traits, parents’ education level, parental self-efficacy, and the impact of problems experienced with the social environment of school) did not contribute to predicting school participation among autistic youths.

**Table 2.** Estimates of the multivariate regression model predicting the school participation of autistic youths with youth factors (i.e. age and autistic traits), family factors (i.e. parents’ education level and parental self-efficacy) and school factors (i.e. the impact of problems experienced with the social and physical environments of school).

	School participation						
	Estimate unstandardized	Standardized	SE	BCa 95% CI^a^ [LL, UL]	Collinearity statistics		*R* ^2^ */ΔR* ^2^
					Tolerance	VIF	
							0.45/0.45*
Youths’ age	1.41*	0.23*	0.65	[0.10, 0.40]	0.98	1.03	
Youths’ autistic traits	−0.14	−0.11	0.14	[−0.20, 0.03]	0.94	1.06	
Parents’ education level	0.05	0.002	2.59	[−0.14, 0.14]	0.97	1.03	
Parental self-efficacy	0.15	0.07	0.23	[−0.10, 0.21]	0.86	1.16	
Impact of problems with the physical environment	−0.58**	−0.64**	0.13	[−0.72, −0.25]	0.52	1.94	
Impact of problems with the social environment	0.02	0.04	0.09	[−0.19, 0.24]	0.47	2.14	

SE: standard error; CI: confidence interval; LL: lower limit; UL: upper limit; VIF: variance inflation factor.

a Estimates of 95% confidence intervals of standardized coefficients using bias-corrected bootstrap (1000 resamples).

* *p* < 0.05, **p < 0.001.

We conducted post hoc analyses to examine whether the weights of the standardized coefficients of the two predictor variables (i.e. autistic youths’ age and problems they experienced with the physical environment of school) differed. Cumming (2009) posits that standardized coefficients would differ significantly if their confidence intervals (CIs) overlapped by less than 50%. To evaluate the extent of overlap (Gignac, 2023), we first calculated the 95% CI for the standardized coefficient of autistic youth’s age (β = 0.23) and problems experienced with the physical environment of school (β = −0.64) (see Table 2), using bias-corrected bootstrap with 1000 resamples. Next, we calculated half of the mean of the overlapping CIs, which yielded a value of 0.14. We added this value to the lower limit (LL) of the CI for the standardized coefficient of autistic youths’ age (LL = 0.25). This yielded a value of 0.39, which was beneath the upper limit (UL) of the CI for the standardized coefficient of problems experienced with the physical school environment (UL = 0.40). This indicates that the overlaps of the CIs slightly exceeded 50% (Gignac, 2023). Consequently, the standardized coefficients of the two predictor variables were considered not to exhibit a statistically significant difference (*p* > 0.05) (Cumming, 2009).

### Pooled results from the imputed data

The correlation analyses using the imputed data showed that school participation was negatively associated with the impact of problems youths experienced with the physical (*r* = −0.34, *p* < 0.001) and social environment of school (*r* = −0.26, *p* = 0.001). In addition, there was a negative association between parents’ education level and youths’ age (*r* = −0.16, *p* = 0.034) and a positive association between the impact of problems experienced with the physical and the social environment of school (*r* = 0.69, *p* < 0.001). These associations remained significant after the Holm-Bonferroni correction. The correlations are reported in Table 3.

**Table 3.** Pooled results: correlations among study variables by Spearman’s rank-order correlation analyses.

	1	2	3	4	5	6
1. School participation	–	–	–	–	–	–
2. Youth age	0.08	–	–	–	–	–
3. Autistic traits	−0.12	−0.04	–	–	–	–
4. Parent education	−0.06	−0.16*	−0.11	–	–	–
5.Parental self-efficacy	0.15	−0.04	0.03	0.03	–	–
6. Impact of problems with the physical environment	−0.34**	0.09	0.10	−0.06	−0.10	–
7. Impact of problems with the social environment	−0.26**	0.07	0.12	0.001	−0.15	0.69**

* *p* < 0.05, **p < 0.001.

When all the predicting variables were entered into the multivariate regression analysis, only one association remained significant: the negative association between autistic youths’ school participation and the impact of problems they experienced with the physical environment at school. See Table 4 for the estimates of the predicting variables.

**Table 4.** Pooled results: estimates of the multivariate regression model predicting the school participation of autistic youths with youth factors (i.e. age, autistic traits), family factors (i.e. parents’ education level, parental self-efficacy) and school factors (i.e. the impact of problems experienced with the social and physical environments of school).

	Estimate unstandardized	SE	*p* Value	95% CI [LL, UL]
Youths’ age	0.17	0.68	0.799	[−1.17, 1.52]
Youths’ autistic traits	−0.26	0.22	0.245	[−0.70, 0.18]
Parents’ education level	−3.2	3.82	0.405	[−10.85, 4.45]
Parental self-efficacy	0.38	0.42	0.376	[−0.50, 1.27]
Impact of problems with the physical environment	−0.55	0.19	0.004	[−0.92, −0.18]
Impact of problems with the social environment	−0.02	0.11	0.855	[−0.24, 0.20]

SE: standard error; CI: confidence interval; LL: lower limit; UL: upper limit.

## Discussion

There has been growing attention to environmental influences on autistic youths’ participation in daily life situations, but there is little quantitative research on the impact of the environment on their school participation. In this study, we investigated associations between youth factors (i.e. age and the level of autistic traits) and environmental factors (i.e. factors related to the family and school environment) on the one hand, and autistic youths’ school participation on the other hand. Using national survey data on 200 autistic youths in the Netherlands, we found that both youth factors and school factors were associated with autistic youths’ participation, revealed by correlation analyses. However, when all factors were added into one regression model, neither the associations with the youth factors nor the association with the impact of problems that autistic youths experienced with the social environment of school maintained statistical significance. The regression model revealed the unique contribution of the impact of problems experienced with the physical environment of school to the prediction of school participation among autistic youths. Furthermore, neither the correlation analyses nor the regression analyses revealed any association between the family factors (i.e. parents’ education level and parental self-efficacy) and autistic youths’ school participation.

Regarding youth-related factors, we expected autistic youths’ age to have a negative association with their school participation. The correlation and regression analyses revealed the opposite: There was a positive association between autistic youths’ age and their school participation. Past research indicated that autistic youths’ adaptive functioning increases with age and autistic symptoms decrease with age (Magiati et al., 2014; Szatmari et al., 2015), and it is these age-related changes that might contribute to increased school participation among autistic youths. Future research adopting a longitudinal approach can better inform us about age-related changes in school participation among autistic youths. Notably, our pooled results, using imputed data, did not reveal a significant association between youths’ age and school participation. This suggests that age-related improvements may not apply uniformly to all autistic youths. Youths with pronounced autistic symptoms and intellectual disabilities, most of whom had missing data and were consequently excluded from the original data analyses, may not experience the same degree of improvement with age as those with milder symptoms and without intellectual disabilities.

In addition to age, we examined whether youths’ autistic traits were negatively associated with their school participation. Initial correlation analyses indicated a negative relation between the level of autistic traits and school participation, but this relation did not maintain statistical significance after correcting for multiple comparisons. In addition, our regression analysis did not reveal a unique contribution of autistic traits to the prediction of school participation. These results are consistent with the findings of previous studies, suggesting that the degree of autistic traits does not inherently hinder autistic youths from participating in daily school activities (Ambrose et al., 2022; Gan et al., 2014; Ryan et al., 2018). As we will discuss later, the barriers to school participation of these youths may primarily stem from factors within the school environment.

As mentioned before, it is crucial to acknowledge that our findings are primarily derived from data of autistic youths with relatively ‘mild’ autism symptoms and without intellectual disabilities. A review study (Russell et al., 2019) highlighted that approximately 50% of the autistic population were estimated to have intellectual disabilities, whereas most autism research, including our study, has been conducted with participants who do not have intellectual disabilities. Consequently, our findings may not be readily applicable to the marginalized group of autistic youths with more pronounced autistic traits and more substantial cognitive challenges.

Regarding family factors, we expected parent education levels and parents’ perceived efficacy in assisting their child with schoolwork to have positive associations with autistic youths’ school participation. However, there were no associations between these family factors and autistic youths’ school participation. This might be explained by the fact that parents who responded to our survey were, by and large, a homogeneous group, with above-average education levels (mean = 2.37 on a scale with the highest possible score of 3; see Table 1) and high self-efficacy (mean = 38.14 on a scale with the highest possible score of 55; see Table 1). It is possible that the group of parents (27.5%) who did not respond to the self-efficacy items have lower confidence and potentially fewer resources available to support their children. Ideally, future research would include families with more diverse socioeconomic backgrounds and greater variation in parent self-efficacy, for a better test of the extent to which these family-related factors play a role in facilitating autistic youths’ school participation.

It is worth noting that previous studies on associations between family socioeconomic status and psychosocial outcomes for autistic youths were mostly conducted in countries where a family’s socioeconomic status can affect an autistic student’s access to medical care and professional support, such as in the United States (e.g. Irvin et al., 2012). In the Netherlands, the healthcare system is relatively equally accessible to all layers of society (Schäfer et al., 2010), so a family’s socioeconomic status may have less influence on psychosocial outcomes for autistic youths (Hrdlicka et al., 2016; Larsson et al., 2005). In addition, the parental self-efficacy we assessed could be more closely related to autistic youths’ academic performances and their educational progress rather than to their participation and involvement in school activities. To better support autistic youths in their daily participation in school activities, which encompasses not only academic pursuits but also social interactions with peers and teachers, it is important to also investigate parents’ contributions to their children’s social learning and the ways in which parents provide emotional guidance and support. These factors may have an impact on the overall school experience of autistic youths.

Regarding school factors, the impact of problems that autistic youths experienced with the physical environment of the school was the only predictor variable (out of all six factors included in this study) that consistently showed a significant effect on predicting autistic youths’ school participation, as revealed by the regression analyses using both the original and imputed data. An accommodating physical environment at school provides youths with a comfortable and safe space to be, and it facilitates their participation in activities. Instead, an unaccommodating physical environment at school imposes barriers, such as a complex building design that hinders a smooth transition between locations, and poor acoustics in classrooms, hallways and canteens where students take breaks and socialize. In many schools, the physical environment often contributes to various kinds of physical discomfort for autistic youths, leading to fatigue, disruptions, and reduced motivation to participate in school activities (Malik et al., 2018; Piller & Pfeiffer, 2017; Rieffe & Koutamanis, 2023).

The other school factor we studied was the impact of problems autistic youths experienced with the social environment of school. We found a negative association between this school factor and autistic youths’ school participation, via the correlation analysis. However, this association was not found in the regression model which accounted for other variables such as the impact of problems experienced with the physical environment of school. Problematic aspects of the physical environment of school might contribute to creating tension between autistic youths and people around them, and hence (partly) accounting for the problematic aspects of the social environment of school. For example, overcrowded canteens, hallways with poor acoustics, and a lack of quiet spaces for reading or private conversations can hinder autistic youths’ engagement with peers. A focus group study revealed that autistic students often sought out empty classrooms during breaks to find solace while their peers socialized with one another (Rieffe et al., 2021). In contrast, modifications to the physical environment of school, such as adjusting the lighting conditions in the classroom (Derakhshanrad & Piven, 2020; Kinnealey et al., 2012), using noise-attenuating devices (Kanakri et al., 2017; Kinnealey et al., 2012), providing clearly defined areas with visible boundaries (Ganz, 2007; McAllister & Maguire, 2012), an escape space for ‘sensory neutralization’ (Mostafa, 2008), and individualized guidance to help autistic students acclimate to the new and unpredictable school environment (Mandy, Murin, Baykaner, Staunton, Cobb, et al., 2016) can foster positive and constructive behaviours in autistic youths. This could in turn increase acceptance and support from teachers and peers, creating a more welcoming social environment for autistic youths to participate in school activities (Dargue et al., 2022; Martin, 2016).

There can be another explanation for the regression model’s insignificant association between the impact of problems experienced with the social environment of school and autistic youths’ school participation. Our study focused on the objective layer of school participation, namely, we asked parents about the extent to which their child participated in educational and social activities at school. We did not explore the subjective layer of school participation, namely youths’ subjective experience while attending school and engaging in school activities (Coster et al., 2012; Imms et al., 2016). However, the subjective layer can be the defining layer of full school participation and the essence of inclusive education (Hodges et al., 2022). While attendance and participation in activities are influenced by how accessible and accommodating the physical environment is, the extent to which autistic youths feel engaged and appreciated may be more closely linked to the acceptance and respectfulness of the social environment (Maxwell et al., 2012). Furthermore, autistic youths may have diverse preferences regarding the types of school activities they wish to engage in, and their desired levels of participation may differ from those of non-autistic youths (Falkmer et al., 2012). A truly inclusive school environment should afford autistic youths as well as those with other educational needs the freedom to choose activities based on their preferences and capacities and allow them to participate in a manner that suits them, rather than imposing rigid routines and uniform requirements on all students.

A strength of this study stems from the use of a national database; we were able to include a large sample of autistic youths of different age groups, with different levels of cognitive and social abilities, from different educational settings, thus increasing the generalizability of the findings of this study. Simultaneously, several study limitations warrant attention.

First, this study addressed just six factors within the individual, family, and school domains. Bölte et al. (2019), on the other hand, identified 27 environmental factors that can influence the functioning of school-aged youths with autism. Among these, the positive attitudes of parents and teachers towards autism (e.g. having a good understanding of the autism condition, expecting autistic youths to participate, and fostering their independence) were found to facilitate autistic youths’ participation in school and leisure activities. Furthermore, barriers in the school system and school policies (e.g. bureaucracy, lack of suitable programming for children with disabilities, segregation of children with disabilities from non-disabled peers) have been found to hinder autistic children’s school participation (Anaby et al., 2013; Gan et al., 2013). The aforementioned factors were not examined in the current study.

Second, the study was based solely on parent-reported information. This is because the data were accessed through the NAR, which gathers information about autistic individuals younger than 16 years only via parents/caregivers. Using data from one informant, the parent or caregiver in this case, introduces a risk of common-method variance bias (Podsakoff et al., 2003). Moreover, parents and caregivers tend to identify fewer problems in the environment than youths themselves (Pivik, 2010). It was also unclear to what extent the parents/caregivers in the current sample were involved in and informed about their child’s school life and school participation. In addition, we do not have information about how many parents have been diagnosed with autism, which could potentially influence their responses to the questionnaires. Ideally, the views of autistic youths and their teachers would be incorporated in a study of school participation, in which some of the factors under investigation pertain to the school environment.

Third, the research team adjusted the CASE questionnaire by editing, adding and removing items to more accurately reflect situations faced by Dutch students and their parents. However, the validity of this modified questionnaire with Dutch samples remains unclear. In addition, the reliability of the scale measuring experienced obstacles in the physical school environment appeared to be low, albeit within an acceptable range (i.e. α = 0.61). Therefore, caution is warranted when interpreting the outcomes related to this scale. Despite this limitation, this study revealed a significant effect of problematic aspects in the physical school environment on autistic youths’ school participation. This underscores the importance of developing standardized measurements that can provide more nuanced insights into the environmental barriers and facilitators affecting autistic youths’ school participation.

The limitations notwithstanding, this study points to the important role of the school environment in relation to autistic youths’ school participation, which has implications for practice and research. First, our findings highlight the importance and urgency of modifying the school environment, to make it more sensitive to the needs of autistic youths, and more accommodating towards their participation. While previous research examining autistic youths’ participation often emphasized personal factors such as the level of autistic traits and socioemotional functioning, our study did not establish a significant association between autistic traits and school participation. This indicates that having autism alone does not necessarily have a negative impact on autistic youths’ school participation. Rather, it might be the interaction between having autism and being placed in an autism-unfriendly school environment that affects autistic youths’ school participation (Bailey & Baker, 2020; Maciver et al., 2023). Second, with respect to research, we suggest that future studies address the subjective layer of autistic youths’ school participation and its contributing factors, increasing our understanding of the quality of autistic youths’ school participation and their lived experience in the school environment (Cappa et al., 2023). This can provide guidance for modifying the social environment of school (e.g. increasing knowledge about autism among school staff and students, promoting positive attitudes towards autism and neurodiversity, reinforcing inclusion at the policy level). Related changes to the social environment of school can help autistic youths feel welcomed, develop a sense of belonging, and enjoy life at school, to the same extent as their non-autistic peers.

## Conclusion

School-aged youths have a basic human right to participate in educational and recreational activities at school. Previous studies indicate that many autistic youths have restricted school participation, and many of them often feel unwelcomed when attending school. Notably, these youths are frequently unfairly held accountable for the exclusion they experience. Recently, there has been a paradigm shift, emphasizing the role of the environment in influencing autistic youths’ full participation in school activities. This study provided new support for the role of the school environment in predicting autistic youths’ school participation. Noteworthy, our findings indicate that problematic aspects of the physical school environment could have a stronger impact on autistic youths’ participation than youth characteristics or family factors. This underscores the importance of modifying the school environment to make it more accommodating for autistic youths’ needs, by creating a comfortable space where these youths can learn and participate to the same extent as their non-autistic peers.

## Supplemental Material

sj-docx-1-aut-10.1177_13623613231225490 – Supplemental material for School participation of autistic youths: The influence of youth, family and school factors Supplemental material, sj-docx-1-aut-10.1177_13623613231225490 for School participation of autistic youths: The influence of youth, family and school factors by Boya Li, David Heyne, Anke Scheeren, Els Blijd-Hoogewys and Carolien Rieffe in Autism
