# Supplementary material for: School participation of autistic youths: The influence of youth, family and school factors
Source: Autism. 2024 Feb 4;28(9):2295–310. doi: 10.1177/13623613231225490 (PMC11408984; doi:10.1177/13623613231225490)
Supplement: sj-docx-1-aut-10.1177_13623613231225490 – Supplemental material for School participation of autistic youths: The influence of youth, family and school factors [file sj-docx-1-aut-10.1177_13623613231225490.docx]

**Supplementary materials**

Supplementary Table 1. Item descriptions (translated from Dutch): Child and Adolescent Scale of Participation (CASP), Child and Adolescent Scale of Environment (CASE), and Parental Self-Efficacy.

| **CASP** |  |
| --- | --- |
|  | There are now 6 questions about how active you child is with others at school. You will be asked to what extent you child participates in the following activities at school, compared to classmates. If your child does not go to school, think then about the last school you child attended. |
|  | 1. Educational activities with classmates at school. |
|  | 2. Social, play, and recreational activities with other students at school (e.g., ‘hanging out’, sports, hobbies, creative activities, lunch, or recess activities). |
|  | 3. Move independently at school (e.g., to the toilet, in the schoolyard, in the library, or to other classrooms). |
|  | 4. Use transportation [bicycle or public transportation] or walk to get to school. |
|  | 5. Use of educational materials and equipment available to classmates or those that might be adapted for your child (e.g., books, computers, chairs, and desks). |
|  | 6. Conversations with other students and adults at school. |
| **CASE** |  |
|  | Here are 10 problems your child may encounter in their daily routine at school. You will be asked how strongly you child feels each problem. If you child does not go to school, think then about the last school your child attended. |
|  | 1. The physical environment at school: It is difficult to get to things and places. |
|  | 2. The physical environment at school: It is difficult to see or hear important information. |
|  | 3. Lack of support and encouragement at school (e.g., from teachers or classmates). |
|  | 4. The attitude of people at school. |
|  | 5. Inappropriate or lack of resources at school. |
|  | 6. Unsuitable or lack of transportation to and from school. |
|  | 7. Inappropriate or lack of help from people at school (e.g., individual guidance from a professional). |
|  | 8. Inappropriate or lack of programs at school (e.g., buddy program or homework help). |
|  | 9. Crime or violence at school. |
|  | 10. Inappropriate or lack of information about your child at school (e.g., information about your child’s development). |
| **Parental self-efficacy** | |
|  | The following are 11 statements about your support for your child at school. You will be asked to what extent you AGREE or DISAGREE with the statement. If you child does not go to school, think then about the last school your child attended. |
|  | 1. I know how to help my child do well in school. |
|  | 2. I don’t know if my child listens to me when I give advice about things at school. |
|  | 3. I don’t know how to help my child get good grades in school. |
|  | 4. If I try my best, I can talk well with my child, even if he/she has trouble understanding something. |
|  | 5. A student’s motivation to do well in school depends on the parents. |
|  | 6. It goes very well when I help my child with learning. |
|  | 7. Other children have more influence on my child’s grades than I do. |
|  | 8. I don’t know how I can help my child with learning. |
|  | 9. I make a big difference in my child’s school performance. |
|  | 10. Whether my child feels like learning depends on what the other children think or do. |
|  | 11. I am good at helping my child learn. |

Supplementary Table 2. Score ranges, means, standard deviations (SD), counts and percentages of missing values for study variables.

| **Variables** | **Missing values** | |
| --- | --- | --- |
|  | **Count** | **Percentage** |
| School participation | 42 | 21 |
| Youths’ age | 1 | 0.5 |
| Youths’ autistic traits | 67 | 33.5 |
| Parent educational level | 11 | 5.5 |
| Parental self-efficacy | 55 | 27.5 |
| Impact problems with the physical environment | 3 | 1.5 |
| Impact problems with the social environment | 3 | 1.5 |
